# Effects of High-Voltage Atmospheric Cold Plasma Treatment on Microbiological and Quality Characters of Tilapia Fillets

**DOI:** 10.3390/foods11162398

**Published:** 2022-08-10

**Authors:** Jiamei Wang, Tengfei Fu, Yuanyuan Wang, Jianhao Zhang

**Affiliations:** 1 College of Food Science and Engineering, Hainan University, Haikou 570228, China; 2 Agricultural Products Processing Research Institute, Chinese Academy of Tropical Agricultural Sciences, Zhanjiang 524000, China; 3 College of Food Science and Technology, Nanjing Agricultural University, Nanjing 210095, China

**Keywords:** cold plasma, tilapia fillet, microbiological, quality

## Abstract

Cold plasma (CP) has become an alternative to conventional thermal processing of food products. In this study, the effect of cold plasma treatment time on the inactivation and quality of tilapia fillets was investigated. The surfaces of tilapia fillets were inoculated with *Salmonella enteritis (S. enteritis), Listeria monocytogenes* (*L. monocytogenes*), and a mixture of both before being treated with cold plasma at 70 kV for 0, 60, 120, 180, 240, and 300 s. With the extension of treatment time, the number of colonies on the surface of the fillets decreased gradually; after 300 s of cold plasma treatment, *S. enteritis* and *L. monocytogenes* populations were reduced by 2.34 log CFU/g and 1.69 log CFU/g, respectively, and the a* value and immobile water content decreased significantly (*p <* 0.05), while the free water content increased significantly (*p <* 0.05). TBARS value increased significantly (*p <* 0.05) to 1.83 mg MDA/kg for 300 s treatment. The carbonyl value and sulfhydryl value of sarcoplasmic protein significantly (*p <* 0.05) increased and decreased, respectively, as treatment time extension, while no significant changes were found in myofibrillar protein. No significant differences were observed in pH, b* value, elasticity, chewiness, thiol value, and TVB-N value. The results showed that cold plasma had an inactivation effect on tilapia fillets and could preserve their original safety indicators. It was concluded that CP treatment could be used as an effective non-thermal method to maintain the quality of tilapia fillets and extend their shelf-life.

## 1. Introduction

Tilapia is popular with its high protein and low fat. China is the largest producer of farmed tilapia in the world, harvesting roughly 1.64 million tons of tilapia in 2019. At present, the main method of storing tilapia fillets is frozen storage (at 18 °C), which is highly energy-consuming and risks water loss during storage [1]. Cold storage is also commonly used for preserving aquatic products where the microbes are relatively active and have a negative impact on the quality of fish fillets [2]. While traditional heat processing methods (cooking, frying, drying, etc.) could sterilize the fish fillet successfully, they caused significant changes in the physical characters and chemical compositions of fish, including the denaturation of protein, decrease in water content, and changes in flavor, thus not suitable for the preservation of aquatic products. Therefore, developing a non-thermal inactivation process is particularly necessary for fish fillets preservation. Over the past decades, several non-thermal technologies have been studied, such as high hydrostatic pressure technology [3], pulsed electric field processing [4], and irradiation [5], which showed high inactivation efficacy with a minor negative influence on the natural quality of fish. However, some of them failed to be widely used in commercial applications due to the high price of equipment or the strict operating conditions [6]. Cold plasma, as an innovative non-thermal treatment, has advantages in terms of low temperature inactivation and operational ease [7]. Although products with high-fat content are easily oxidized by the active substances produced during cold plasma treatment [8], cold plasma treatment has a relatively broader prospect in researching tilapia fillets with low-fat content.

The research on cold plasma treatment of food has been widely reported [9]. Various reactive species such as reactive nitrogen species (RNS), reactive oxygen species (ROS), energetic ions, ultraviolet (UV) radiation, and charged particles generated during cold plasma have been reported as antibacterial agents [10]. It has been proved that with active substances that can destroy the cell membrane, DNA, and proteins of the bacteria, cold plasma can achieve high efficiency of inactivation [11,12]. In the study of herring (*Clupea harengus*) treated with a DBD cold plasma system, compared to the control samples, there were significant decreases in total aerobic mesophilic bacteria, total aerobic psychrotrophic bacteria, lactic acid bacteria, *Pseudomonas,* and *Enterobacteriaceae* [13]. When mackerel was treated with DBD cold plasma at 70 kV and 80 kV for 1, 3 and 5 min, respectively, the spoilage bacteria (total aerobic psychrotrophic bacteria, *Pseudomonas,* and lactic acid bacteria) on the surface of mackerel were significantly reduced [14]. The total aerobic psychrotrophic bacteria in the Asian sea bass decreased significantly after cold plasma treatment, which effectively extended the shelf life of the fish [15]. In addition, in the study of semi-dried Pacific saury treated by corona discharge plasma jet, cold plasma not only had a significant inactivation effect on total aerobic psychrotrophic bacteria, *Staphylococcus aureus,* and other bacteria, but also caused the number of mold and yeast to decrease significantly [16]. In terms of fish quality, lipids oxidation [14] and protein oxidation [15] of fish have been found to be the results of cold plasma treatment. Interestingly, it was reported that the cold plasma treatment had no significant effect on lipids oxidation of Atlantic mackerel, but it accelerated the oxidation of protein [17]. Additionally, the influence of cold plasma on *Scomber Japonicus* quality was demonstrated from the determination of TVB-N value, TBARS value, and PV value, respectively [18]. Similarly, in the study of Pacific White Shrimp (Litopenaeus Vannamei), the content of chemical indexes, including pH, TVB-N, TBARS, and PV, reflected the influence of cold plasma on the quality of Shrimp [19].

A search of the literature revealed few studies had been conducted on cold plasma treatment of tilapia at the present stage. Therefore, in this study, the material was the peeled tilapia fillet which was treated with cold plasma excited by air. The inactivation effect of cold plasma on the fish fillet inoculated with *Salmonella enteritis (S. enteritis)* and *Listeria monocytogenes (L. monocytogenes)* was evaluated. The quality indicators of fish fillet treated by cold plasma were evaluated by measuring the color, pH, TBARS level, carbonyl level, sulfhydryl level, total volatile basic nitrogen, texture, and low field NMR analysis.

## 2. Materials and Methods

The study was conducted from October 2020 to August 2021.

### 2.1. Sample Preparation

The fresh tilapia fillets were purchased from Hainan Quanyi Foods Co., Ltd. (Haikou, China). The fresh tilapias were put on ice and transported to the laboratory as soon as possible after being peeled, boned, and cleaned at the factory. The tilapia fillets were irradiated by an ultraviolet lamp before inoculation.

*S. enteritis* (21635) and *L. monocytogenes* (24119) were purchased from CICC (China Center of Industrial Culture Collection, China). *S. enteritis* and *L. monocytogenes* were cultivated in 100 mL nutrient broth and brain heart infusion broth at 37 °C for 10 h, respectively. Bacterial cells were harvested by repeating the centrifugation (6000 r/min for 15 min) at 4 °C and rinsing twice with sterile phosphate-buffered saline (PBS, pH 7.4). The cells were resuspended in sterile PBS to achieve a concentration of about 7 log CFU/mL.

For single bacterium inoculation, the fillets were soaked in *S. enteritis* and *L. monocytogenes* suspension for 1 min, respectively. After inoculation, the tilapia fillets were dried in a sterile and ventilated environment (30 min) and then placed in PP food packaging boxes sealed by a modified atmosphere packaging machine (MAP-H360, Senrui Fresh-keeping Equipment Ltd., Suzhou, China).

For mixture bacteria inoculation, the irradiated fillets were soaked in a mixed suspension for 1 min (*S. enteritis*: *L. monocytogenes* ratio of 1:1 (*v/v*)). After inoculation, the tilapia fillets were packaged using the same method as mentioned above.

### 2.2. Cold Plasma Treatment

In this study, A dielectric barrier discharge (DBD) cold plasma system was used for treatment as described by Wang, Zhuang, Lawrence, and Zhang [20]. The packaged tilapia fillets were treated by a DBD system at 70 kV voltage for 60 s, 120 s, 180 s, 240 s, and 300 s. Control groups without treatment were carried out at the same time.

### 2.3. Microbiological Analysis

The samples (5 g) were put into a sterile homogenizer containing 45 mL of 0.85% sterile saline and then flapped for 2 min using a slap homogenizer to make 1:10 sample diluents. After homogeneous mixing, the suspension was diluted serially with 0.85% sterile saline, and then appropriate dilutions of 0.1 mL were inoculated on solid media plates. The PCA (plate count agar) (Hopebio, Qingdao, China) was used for single bacterial inoculation samples. GNBSMs (Gram-Negative Bacteria Selective Medium) (Hopebio, Qingdao, China) and MMAs (Modified Mcbride Agar Base) (Hopebio, Qingdao, China) were used for mixed bacterial inoculation samples. GNBSMs were used as a selective media for *S. enteritidis*. Based on the introductions of media, 1 mL sterile penicillin solution (including penicillin G96 unit) (Hopebio, Qingdao, China) was added to a 200 mL sterile medium (45–50 °C) before the agar culture cooled. MMAs were used as the selective culture of *L. monocytogenes*; 3 mg of Fudaxin (Hopebio, Qingdao, China) was added to 100 mL sterile medium (50–55 °C) before the agar culture cooled. The agar plates were incubated at 37 °C for 48 h, and the colonies were counted and reported as log CFU/g. The plates without visible growth were incubated for 72 h to confirm the absence of colonies.

### 2.4. pH Analysis

The samples (5 g) were homogenized using 45 mL saturated KCl solution for 1 min, and then, the pH value of the resulting homogenate was determined using a pH meter (REX, pHS-3C, Shanghai, China).

### 2.5. Color Analysis

The color of fillets was determined using a color reader (CR-10, Konica Minolta, Japan). The manufacturer’s standard white plate was used for calibration. The lightness (L*), red/green (a*), and yellow/blue (b*) were measured.

The color change (ΔE) was calculated as:

$$\Delta E=\sqrt{{\left(L_{t}-L_{0}\right)}^{2}+{\left(a_{t}-a_{0}\right)}^{2}+{\left(b_{t}-b_{0}\right)}^{2}}$$

The chromaticity was calculated as:

$$\Delta C=\sqrt{{\left(a_{t}-a_{0}\right)}^{2}+{\left(b_{t}-b_{0}\right)}^{2}}$$

where subscript 0 denotes the initial color value of the fillet, and subscript t denotes the color value of the fillet after cold plasma treatment.

### 2.6. Low Field NMR (Nuclear Magnetic Resonance) Analysis

The samples (15 g) were placed in a 40 mm MRI tube and tested at 25 °C. The Low Field Magnetic Resonance Imaging Analyzer (NM120-040H-I, NIUMAG, Suzhou, China) with a resonance frequency of 21.3 MHz was used to generate NMR data. The Carr–Purcell–Meiboom–Gill (CPMG) pulse sequence was used for lateral measurement (T_2_). The instrument was based on the SIRT algorithm with its own software version 4.0 (NIUMAG INSTRUMENT), and the T_2_ spectrum was obtained using inversion when the iteration time was 100,000.

### 2.7. Texture Profile Analysis (TPA)

The TPA of fish fillets was determined using a texture analyzer (CT3, AMETEK. Inc., Brookfield, WI, USA) with a cylindrical probe (6 mm in diameter) at a test speed of 4 mm/s and a test distance of 2 mm. Each slice of fish was measured at three points, and the results were averaged.

### 2.8. Thiobarbituric Acid Reactive Substances (TBARS) Analysis

Lipids oxidation was measured by the method described by Huang, Wang, Zhuang, Yan, and Zhang [21] with little modification. Five grams of fish fillet were homogenized with 15 mL of 7.5% trichloroacetic acid (TCA) solution at 10,000 rpm for 30 s. The mixture was filtered, and 10 mL filtrate was mixed with an equivalent 20 mM TCA solution in a tube. Then, the tube was kept in a boiling bath for 30 min. The absorbance of the solution was read in a spectrophotometer (UV-2450, Shimadzu Co., Kyoto, Japan) after cooling. The concentration of TBARS was expressed as mg MDA (malondialdehyde) per kg of fish fillets.

### 2.9. Protein Carbonyls and Sulfhydryls Analysis

Sarcoplasmic protein and myofibrillar protein of tilapia fillets were extracted according to the method proposed by Toldra, Rico, and Flores [22]. Total carbonyls were measured by the method described by Mesquita et al. [23]. The 0.4 mL of protein solution was mixed with a 0.4 mL 10 mmol/L 2,4-dini-rophenylhydrazine (DNPH) solution (containing 0.5 mol/L H_3_PO_4_). The mixed solution was reacted in the dark at 25 °C for 10 min. Then, 0.2 mL 6 mol/L NaOH solution was added to the mixture and kept at 25 °C for 10 min. The absorbance of the mixture solution was measured at 450 nm in a spectrophotometer (JC-721, Qdjuchuang, Qingdao, China). Protein concentration was determined with a protein kit (Nanjing Jiancheng Biological Engineering Institute, Nanjing, China). Carbonyl concentration was expressed as nmol per mg protein.

Protein sulfhydryls were measured using Ellman’s reagent in accordance with the method described by Srinivasan and Hultin [24]. The protein concentration was adjusted to 2 mg/mL with PBS buffer, 0.5 mL of the diluted protein solution mixed with 2 mL urea-sodium dodecyl sulfate (SDS) solution (containing 8.0 mol/L urea, 30 g/L SDS, 0.1 mol/L sodium phosphate buffer, pH 7.4) and 0.5 mL 10 mmol/L 2-nitrobenzoic acid (DTNB) reagent (dissolved in 0.1 mol/L sodium phosphate buffer, pH 7.4), The mixture was kept at room temperature for 15 min, then the absorbance value of supernatant was measured at 412 nm in a spectrophotometer (JC-721, Qdjuchuang, Qingdao, China). The results were expressed as mol per g protein.

### 2.10. Total Volatile Basic Nitrogen (TVB-N) Analysis

TVB-N value was determined using the method proposed by Cao et al. [25]. The fish sample was left to spread in 10 times of water for 30 min. The content of TVB-N was determined using a Kjeldahl apparatus (KDy-9820, Beijing, China) and titrated by 0.01 M HCl.

### 2.11. Statistical Analysis

The SPSS software (SPSS 20.0 for Windows, SPSS Inc., Chicago, IL, USA) was employed for data analysis. Data obtained from the experiment run in triplicates were subjected to one-way analysis of variance (ANOVA) and Duncan’s multiple range test, and *p*-values less than 0.05 were considered statistically significant.

## 3. Results and Discussion

### 3.1. Bacterial Analysis

**Figure 1:**
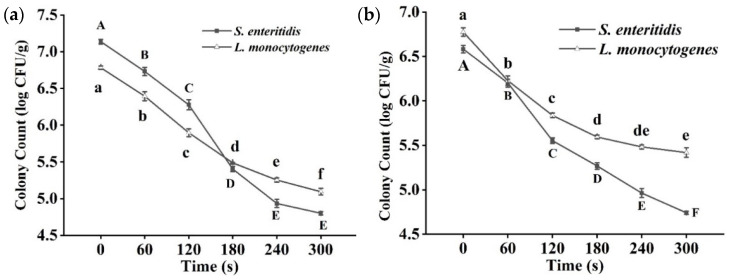
(**a**) Changes in bacteria numbers (individual inoculation) of tilapia fillets after cold plasma treatment. (**b**) Changes in bacteria numbers (mixed inoculation) of tilapia fillets after cold plasma treatment. Different letters indicate significant changes with changes in treatment time.

The effects of cold plasma on microorganisms are affected by the surface characteristics of the product. Potential irregularities on the surface of fish, including cracks, grooves, and pits, may have a blocking effect on the active material derived from cold plasma. The effect of cold plasma treatment time on the inactivation of tilapia fillet was investigated using 70 kV as the treatment voltage. When *S. enteritis* and *L. monocytogenes* were separately inoculated on fillets, after 300 s of cold plasma treatment, *S.* *enteritis* and *L. monocytogenes* populations were reduced by 2.34 log CFU/g and 1.69 log CFU/g, respectively (Figure 1a). The population of surviving bacteria of both strains decreased significantly (*p <* 0.05) with the extension of treatment time. Regardless of whether the fillet was inoculated with Gram-negative bacteria (*S. enteritis*) or Gram-positive bacteria (*L. monocytogenes*), the decrease in the population of bacteria was time-dependent after cold plasma treatment. In a mixed inoculation test, under the same cold plasma treatment conditions, the populations of *S. enteritis* and *L. monocytogenes* in tilapia fillet decreased by 1.84 log CFU/g and 1.36 log CFU/g, respectively (Figure 1b). The antibacterial efficiency of cold plasma against the bacteria in the mixture was weaker in comparison with the single bacterial inoculation test, probably because the mixed bacteria had a synergistic resistance reaction to cold plasma. In the treatment of beef jerkies inoculated with *L. monocytogenes* and *Staphylococcus aureus*, cold plasma had an excellent inactivation effect [26].

It is noteworthy that cold plasma has a better inactivation effect on G^−^ than G^+^ bacteria inoculated separately or in combination, and many studies have reported similar results [12]. This may be due to the inactivation mechanism and the differences between the structures of G^−^ and G^+^ bacteria; G^−^ bacteria have thinner cell walls, which are similar to cell membranes, with a double-membrane composed of unique lipopolysaccharides, phospholipids, and proteins. G^+^ bacteria cell walls contain a peptidoglycan layer, which is much thinner than that in G^−^ bacteria [27]. It was found that due to the difference in cell walls, the leakage content of intracellular substances from G^−^ bacteria was more serious than that of G^+^ bacteria after being treated with cold plasma [28]. On the other hand, charged particles, reactive oxygen species, reactive nitrogen radicals, and other active substances that are produced by cold plasma using air as the reaction medium are determining factors of inactivation efficiency [29]. They react directly with the outer membrane of G^−^ bacteria, while G^+^ bacteria need to enter the cell before they can react with intracellular substances [12].

### 3.2. pH Analysis 

**Figure 2:**
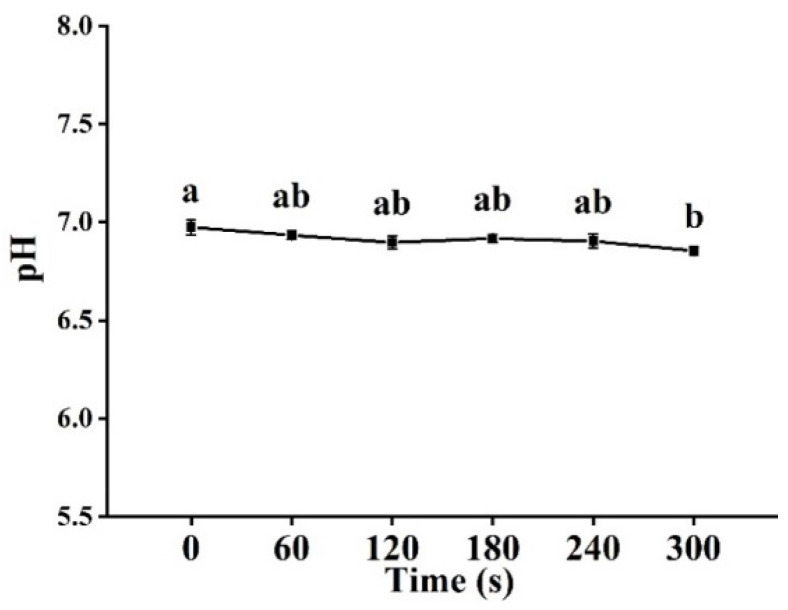
Changes in pH value of tilapia fillets after cold plasma treatment. Different letters indicate significant changes with changes in treatment time.

The pH value is a reliable indicator of fish quality. The influence of cold plasma treatment on the pH value of tilapia fillet is shown in Figure 2. No significant difference in pH values was observed between tilapia fillet samples until they were treated for 300 s. Compared with the control group, the pH value of fish fillet treated for 300 s decreased from 6.97 to 6.85 (*p <* 0.05), similar results were reported in the studies of herring [13] and pork loin [30]. It was found that the decrease in pH value in cold plasma treatment was mainly due to the formation of acidic substances, such as NO_X_, which mainly appeared in oxygen-containing multi-reaction systems [31].

### 3.3. Color Analysis

Color is a prominent feature of fish, which directly affects consumers’ acceptance. The influence of cold plasma treatment time on tilapia fillet color is shown in Table 1. The ΔE increased gradually after cold plasma treatment, which proves that the color change increased with the increase in treatment time. The chromatic value of tilapia fillet was related to a* value and b* value, increasing significantly after treatment. With the increase in cold plasma treatment time, the L* value of tilapia fillet increased, and a* value decreased (Table 1). The control group had the lowest L* value and the highest a* value, while the values of samples treated with cold plasma for 300 s showed the opposite trend. No significant difference in b* value was observed between the treatment group and the control group. It is possible that lipids oxidation resulted in changes in color parameters.

L* value of cold plasma treated chicken breast increased with the extension of treatment time [32]. It has been reported that there was no significant change in b* value after plasma treatment, regardless of the type of meat, including fish [14], chicken [33], and pork [34], which was similar to the results of this experiment. The a* value, which has the greatest effect on sensory perception, has been reported to decrease significantly in fresh pork after cold plasma treatment [35]. The color change of red meat is related to the oxidation of myoglobin (Mb); Mb is a binding protein composed of a peptide chain and a heme prosthetic group. Oxymyoglobin gives meat its bright red [36]. Our results of tilapia fillet research also confirmed this opinion. After exposure to strong oxides produced by cold plasma, the fish meat oxidized, the metmyoglobin formed, and the color became darker.

### 3.4. Low Field NMR Analysis

**Figure 3:**
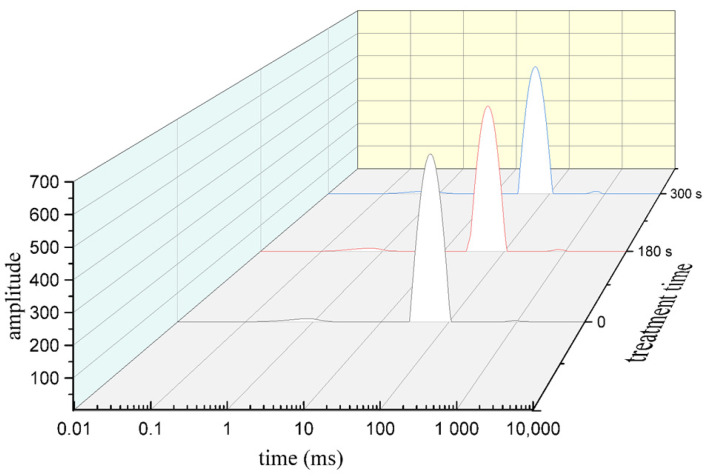
Low field NMR distribution of relaxation times of tilapia fillets after cold plasma treatment.

Moisture content and moisture distribution change dynamically during the whole process of meat processing and storage, which are important factors that determine fish meat quality and shelf life. Useful information on the interaction between water and myofibril was provided by low field NMR (Figure 3). Three peaks appearing in the spectrum were considered to be directly related to the three water components in muscle tissue, i.e., bound water, immobilized water, and free water. Bound water is very closely associated with biomacromolecules in tilapia fillets and is almost impossible to remove. T21 represented immobilized water located in a protein-dense myofibril network. Free water is also known as myofibril external water (T22). Most of the water detected in all the treatment conditions was immobilized water with a content of 95% and above. Low field NMR was performed for tilapia fillets treated with cold plasma in groups for 0 s, 180 s, and 300 s (Table 2). After cold plasma treatment, the content of immobilized water decreased significantly (*p <* 0.05), while the content of free water increased significantly (*p <* 0.05). Interestingly, from the relaxation time of low field NMR analysis in Figure 3, it can be observed that the relaxation time of free water moved forward slightly after cold plasma treatment. In the experiment of cold plasma treatment of mackerel, the results of low-field NMR analysis of moisture distribution in fish showed that the content of T21 immobilized water decreased significantly [14], which was consistent with the results of this experiment. It was speculated that the change in muscle fiber structure was related to immobilized water. At the same time, water holding capacity was also an important index for observing the change in water content. It was reported that the water holding capacity of chicken breast meat decreased significantly after cold plasma treatment at 100 kV for 5 min [37]. Studies have shown that cell membranes are damaged after being treated with cold plasma, which may be one of the causes of the loss of immobilized water in fish [38]. In addition, the denaturation of protein on the surface of fish must not be overlooked. Due to the changes in protein structure as a result of cold plasma treatment, the content of moisture bound to the protein will not remain the same.

### 3.5. Texture Analysis

The texture is one of the most important sensory factors for consumers of meat products, which can be used to evaluate the quality of the product. The hardness of the fish fillet decreased gradually after cold plasma treatment (Table 3). When samples were treated for 120 s, the hardness decreased much faster, and it decreased to 224.20 g after 300 s treatment. There was a significant difference in the chewiness of fish fillets between treatment and control groups, but no significant difference was observed between treatment groups. Nor was there any significant difference in the elasticity of cold plasma treated tilapia fillet. In terms of the viscosity and cohesion of tilapia, only the fish treated for 300 s showed significant differences from other factors.

A recent study [39] showed that cold plasma treatment had no significant effect on the texture of Asian bass. In the study of pork and beef treated with cold plasma, with the extension of treatment time, there was no significant difference in the hardness, elasticity, and chewability of pork and beef compared with the control group [35]. A similar result of elasticity was found in this study. The decrease in the hardness of fish may be related to the loss of immobilized water. In addition, only a slight difference between the fish before and after cold plasma treatment was observed in the elasticity, chewiness, viscosity, and cohesion, which means the whole structure of tilapia fillet was not significantly damaged by cold plasma.

### 3.6. TBARS Analysis

**Figure 4:**
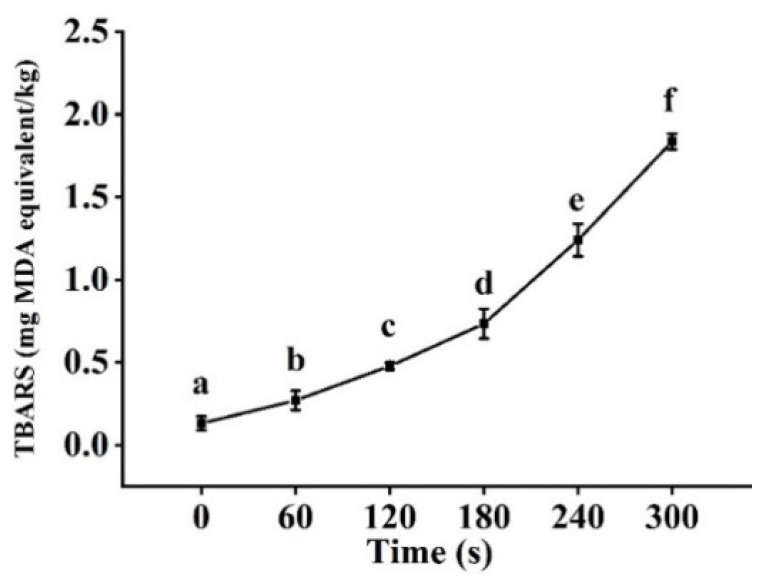
Changes in TBARS value of tilapia fillets after cold plasma treatment. Different letters indicate significant changes with changes in treatment time.

After cold plasma treatment, the TBARS value of tilapia fillet increased significantly (*p <* 0.05), especially after 180 s of treatment (Figure 4). When samples were treated for 300 s, the TBARS value increased to 1.83 mg MDA/kg but far below 8.0 mg MDA/kg, which was considered the acceptable limit for aquatic food [40,41].

The effect of cold plasma treatment on the TBARS level of meat has been demonstrated in a number of studies. In the studies of Bae, Park, Choe, and Ha [42], with the extension of exposure to atmospheric jet plasma, the TBARS levels of fresh beef loin, pork shoulder, and chicken breast increased significantly, and all of them were lower than 1.0 mg MDA/kg, which was within the acceptable range. A large number of prooxidants (RNS and ROS) were produced by atmospheric cold plasma, and the production of lipid oxidation by-products such as hexanal and malondialdehyde were formed during the reaction of ROS and fatty acids [43]. However, in the cold plasma treatment using argon as the reaction gas, the TBARS level did not change significantly in meat after cold plasma treatment, which indicated that the TBARS value was affected by the reaction medium gas [26].

### 3.7. Carbonyl and Sulfhydryl Level Analysis

**Figure 5:**
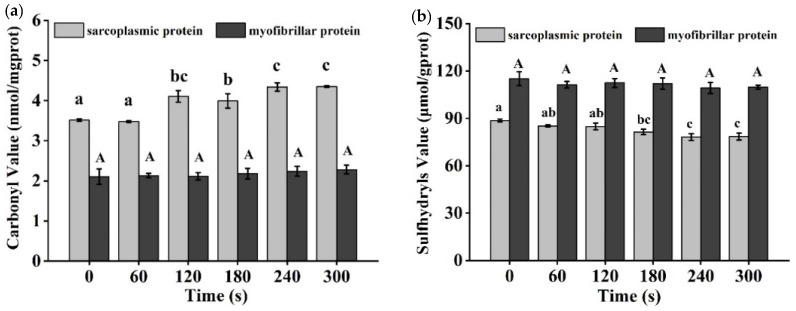
(**a**) Changes in carbonyl values of tilapia fillets after cold plasma treatment. (**b**) Changes in Sulfhydryl values of tilapia fillets after cold plasma treatment. Different letters indicate significant changes with changes in treatment time.

As shown in Figure 5a, the level of protein carbonyl in tilapia fillets varied as cold plasma treatment time changed. The carbonyl level of the sarcoplasmic protein did not change significantly in 60 s of treatment, and it showed an increasing trend accompanied by a slight fluctuation until it finally reached 4.2 nmol/mg prot when the treatment time was longer than 120 s. However, the carbonyl of myofibrillar protein was maintained at a low level, and no significant change in carbonyl level was observed in treated samples.

It was reported that the total carbonyl content in cold-plasma-treated Asian bass was significantly higher than that in the control group [44]. The oxidation of protein side chains is an important factor in the formation of carbonyl groups [45]. In addition, protein structures and peptide chains are vulnerable to cold plasma treatment, and peptide bond cleavage may result in the exposure of amino acid groups [46]. There are so many kinds of strong oxidizing substances in atmospheric cold plasma that can fully react with protein. Slight oxidation occurred in whey protein isolate (WPI) after 15 min of atmospheric cold plasma treatment, which changed the side chain of the amino acid residues and increased the carbonyl content [47].

The sulfhydryl groups of cysteine residues in proteins are extremely susceptible to oxidation induced by most forms of ROS, which provides an additional indicator of the level of protein oxidation. In all of the samples treated with cold plasma, the sulfhydryl content of sarcoplasmic protein decreased slightly to 78.51 mol/gprot after 300 s of treatment (Figure 5b). The sulfhydryl content of myofibril protein decreased slightly after cold plasma treatment.

After 300 s of cold plasma treatment with crude protease extracted from hairtail fish, sulfhydryl content decreased significantly (*p <* 0.05) compared with the control group [48]. Miao et al. [49] found that the sulfhydryl levels of myofibril extract from Alaskan cod decreased significantly after treatment with dielectric barrier discharge cold plasma. They considered that the reduction of sulfhydryl content was the result of their reaction with reactive oxygen species. Since sulfhydryl is involved in the formation of disulfide bonds, the reduction of disulfide bond formation has a significant impact on protein folding and stability. In the study of shrimp treated with plasma-activated water, no significant change in myosin sulfhydryl of shrimp was found [50], which was similar to the result of the tilapia research in this study. Protein oxidation induced by cold plasma treatment occurs easily in the extracted protein solution or protein model systems, and significant differences only occur after the directly treated foods are stored for a long period [51].

### 3.8. TVB-N Analysis

**Figure 6:**
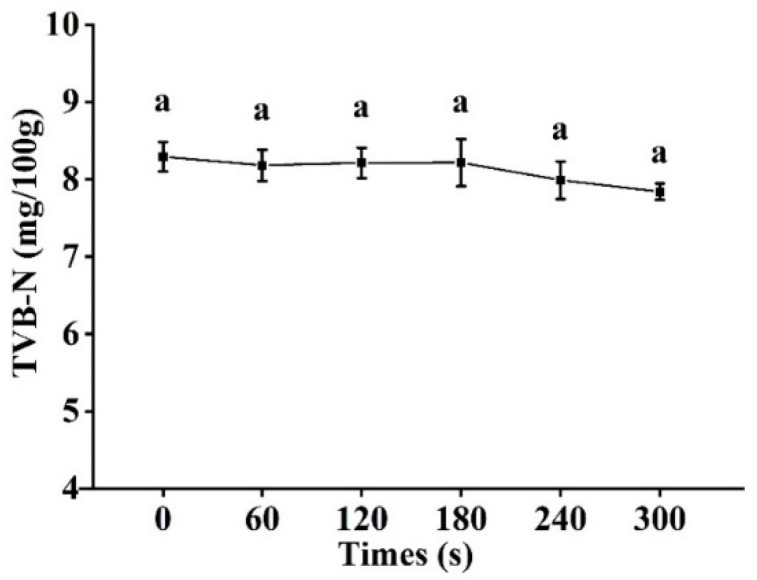
Changes in TVB-N value of tilapia fillets after cold plasma treatment. Different letters indicate significant changes with changes in treatment time.

The TVB-N value of meat is usually the biomarker of bacterial and enzymatic degradation of protein and non-protein nitrogenide, including volatile ammonia, methylamine, dimethylamine, trimethylamine, and other low-level amines. The content of TVB-N is usually used as an indicator of fish freshness. In this study, no significant changes in TVB-N values of tilapia fillets were observed after cold plasma treatment (Figure 6).

In this study, the TVB-N values varied almost identically to the pH values. Zouelm, Abhari, Hosseini, and Khani [19] found that the TVB-N values in cold plasma treated samples kept to similar levels throughout the storage period. The acidic components produced during the plasma treatment can react with the alkaline components, resulting in a reduction of measurable alkaline substances and a lower TVB-N value. Meanwhile, due to its antibacterial effect, the cold plasma treatment could prevent protein breakdown in the meat and hinder the production of alkaline compounds, thus reducing the TVB-N value [18].

## 4. Conclusions

The inactivation effect of cold plasma on inoculated tilapia fillets was increased with the extension of treatment time and a better bactericidal effect of single bacteria strain than a mixture. As the treatment extended, the TBARS increased significantly (*p* < 0.05), which means that the longer time treatment enhanced the lipids oxidation. Sarcoplasmic protein was also oxidation during treatment processing. Therefore, the lipids and sarcoplasmic protein in tilapia fillets were sensitive to oxidation during treatment. Controlling treatment conditions would be helpful in limiting the oxidation degree of lipids and protein. In conclusion, cold plasma treatment could inhibit the growth of bacteria on fish surfaces without a significant negative influence on the organoleptic quality of tilapia fillets. Therefore, cold plasma is a promising alternative to traditional antibacterial treatments used in perishable seafood products.

## Figures and Tables

**Table 1:** Effect of cold plasma treatment on color of tilapia fillets.

Time (s)	ΔL	Δa	Δb	ΔE	ΔC
60	2.02 ± 0.43 ^ab^	−1.03 ± 0.15 ^b^	0.07 ± 0.18 ^ab^	2.30 ± 0.41 ^a^	1.06 ± 0.16 ^a^
120	2.13 ± 0.57 ^b^	−1.93 ± 0.09 ^bc^	0.90 ± 0.14 ^ab^	3.08 ± 0.37 ^a^	2.14 ± 0.14 ^ab^
180	3.85 ± 0.09 ^c^	−3.07 ± 0.53 ^c^	0.88 ± 0.60 ^ab^	5.08 ± 0.47 ^b^	3.25 ± 0.67 ^bc^
240	5.07 ± 0.20 ^d^	−3.86 ± 0.56 ^d^	0.34 ± 0.18 ^b^	6.38 ± 0.17 ^c^	3.87 ± 0.56 ^c^
300	5.37 ± 0.27 ^d^	−4.04 ± 0.32 ^d^	0.11 ± 0.22 ^ab^	6.72 ± 0.41 ^c^	4.04 ± 0.32 ^c^

Different letters in same column indicate significant changes with changes in treatment time.

**Table 2:** Effect of cold plasma treatment on water content in different states of tilapia fillets.

Time (s)	T_2b_	T_21_	T_22_
0	62.95 ± 3.42 ^a^	2002.15 ± 27.43 ^a^	8.47 ± 1.97 ^a^
180	64.55 ± 3.88 ^a^	1946.55 ± 18.97 ^ab^	11.72 ± 1.39 ^a^
300	64.26 ± 4.28 ^a^	1925.85 ± 10.41 ^b^	15.23 ± 1.84 ^b^

Different letters in same column indicate significant changes with changes in treatment time.

**Table 3:** Changes in texture of tilapia fillets after cold plasma treatment.

Time (s)	Hardness (g)	Elasticity (mm)	Viscous (g)	Chewiness (mJ)	Cohesion
0	594.50 ± 21.50 ^a^	2.96 ± 0.09 ^a^	2.14 ± 0.34 ^a^	6.77 ± 0.41 ^a^	0.44 ± 0.02 ^a^
60	373.80 ± 19.70 ^ab^	2.89 ± 0.05 ^a^	2.43 ± 0.37 ^a^	5.08 ± 0.27 ^b^	0.47 ± 0.01 ^ab^
120	327.80 ± 18.90 ^b^	2.96 ± 0.05 ^a^	2.57 ± 0.29 ^ab^	4.87 ± 0.19 ^b^	0.49 ± 0.02 ^ab^
180	283.70 ± 10.50 ^b^	2.93 ± 0.02 ^a^	2.71 ± 0.30 ^ab^	4.75 ± 0.46 ^b^	0.49 ± 0.01 ^ab^
240	257.20 ± 13.40 ^c^	2.89 ± 0.05 ^a^	3.14 ± 0.51 ^ab^	4.25 ± 0.44 ^b^	0.51 ± 0.02 ^b^
300	224.20 ± 10.20 ^d^	2.99 ± 0.08 ^a^	3.86 ± 0.59 ^b^	4.13 ± 0.31 ^b^	0.51 ± 0.01 ^b^

Different letters in same column indicate significant changes with changes in treatment time.

## Data Availability

Data is contained within the article.
